# Influence of postural changes on haemodynamics in internal carotid artery bifurcation aneurysm using numerical methods

**DOI:** 10.1186/s42492-022-00107-2

**Published:** 2022-04-08

**Authors:** Raghuvir Pai Ballambat, Mohammad Zuber, Shah Mohammed Abdul Khader, Anurag Ayachit, Kamarul Arifin bin Ahmad, Rajanikanth Rao Vedula, Sevagur Ganesh Kamath, Ibrahim Lutfi Shuaib

**Affiliations:** 1 Department of Mechanical and Industrial Engineering, Manipal Institute of Technology, Manipal Academy of Higher Education, Manipal, 576104 India; 2 Department of Aeronautical and Automobile Engineering, Manipal Institute of Technology, Manipal Academy of Higher Education, Manipal, 576104 India; 3 Department of Radiology and Imaging, Kasturba Medical College, Manipal Academy of Higher Education, Manipal, 576104 India; 4 Department of Aerospace Engineering, Faculty of Engineering, Universitist Putra Malaysia, 43499 Kuala Lumpur, Malaysia; 5 Department of Cardio-Vascular and Thoracic Surgery, Kasturba Medical College, Manipal Academy of Higher Education, Manipal, 576104 India; 6 Advanced Medical and Dental Institute, Universiti Sains Malaysia, 13200 George Town, Malaysia

**Keywords:** Carotid aneurysm, ANSYS fluid–structure interaction, Altered gravity, Haemodynamics

## Abstract

Cerebral intracranial aneurysms are serious problems that can lead to stroke, coma, and even death. The effect of blood flow on cerebral aneurysms and their relationship with rupture are unknown. In addition, postural changes and their relevance to haemodynamics of blood flow are difficult to measure in vivo using clinical imaging alone. Computational simulations investigating the detailed haemodynamics in cerebral aneurysms have been developed in recent times not only to understand the progression and rupture but also for clinical evaluation and treatment. In the present study, the haemodynamics of a patient-specific case of a large aneurysm on the left side internal carotid bifurcation (LICA) and no aneurysm on the right side internal carotid bifurcation (RICA) was investigated. The simulation of these patient-specific models using fluid–structure interaction provides a valuable comparison of flow behavior between normal and aneurysm models. The influences of postural changes were investigated during standing, sleeping, and head-down (HD) position. Significant changes in flow were observed during the HD position and quit high arterial blood pressure in the internal carotid artery (ICA) aneurysm model was established when compared to the normal ICA model. The velocity increased abruptly during the HD position by more than four times (LICA and RICA) and wall shear stress by four times (LICA) to ten times (RICA). The complex spiral flow and higher pressures prevailing within the dome increase the risk of aneurysm rupture.

## Introduction

Computational haemodynamics has become a powerful and desirable tool in the investigation of cardiovascular diseases, such as aneurysm and atherosclerosis, as observed in several recent studies [1]. The altered haemodynamics in these cardiovascular diseases influence the progression of the disease and arterial deformation, and also significantly change the regional blood rheology. Aneurysms are anatomically defined as local dilatations or ballooning of the vessel wall, and they commonly occur in cerebral vessels or near the aorta (aortic aneurysm). Arteriosclerotic diseases, head injuries, severe infections are some of the prominent reasons for aneurysms formation and its growth. However, beyond certain size, aneurysms can rupture due to hydrocephaly, cerebral vascular spasm, intracranial hematoma, subsequent recurrent bleeding resulting in serious complications [2]. The critical criterion in aneurysm treatment is the aneurysm size. Some of the imaging modalities such as computed tomography scanning and magnetic resonance imaging are widely used to diagnose aneurysms and upon detection. They are evaluated to understand the mechanism and risk of treatment versus risk of rupture [3]. The available treatment possibilities include gold standard procedure like surgical clipping, endovascular coil embolization and stenting. However, among various treatment options available, treatment choice mainly depends on individual risk assessment of each aneurysm, including the aneurysm-like location, size, and shape [4]. Haemodynamics in aneurysms is highly influenced by its shape, geometry, sac volume, aspect ratio and relation to the parent vessel [5]. Some of the haemodynamics factors which have vital role in evaluating the pathogenesis of aneurysms and thrombosis are blood velocity, sac pressure, blood velocity and flow impingement. These factor also helpful in understanding the outcomes of endovascular and surgical interventions. However, it is difficult to measure these haemodynamic quantities *in vivo*, while various modeling approaches and numerical simulations can be useful in evaluation of these measurements using fluid–structure interaction (FSI) have been considered in the past [6]. Numerical simulations on cardiovascular system provides valuable information to clinical doctors. These simulations of blood flow in large arteries enhances information from imaging modalities and help doctors to provide better personalized treatment decisions [7, 8].

As fundamental understanding, the blood pressure (BP) in cardiovascular system of human body consists of (1) dynamic pressure, (2) mean systemic filling pressure and (3) hydrostatic pressure [9]. The hydrostatic pressure and dynamic pressure generated during pumping of heart together counts to the total pressure in any particular blood vessel. Hence, the effect of hydrostatic pressure on large arteries cannot be neglected during various postural changes.

Several clinical and experimental studies have demonstrated changes in blood flow behavior during postural changes. Clinical study was more focused on understanding and demonstrating the significant variation of blood flow in various postures. The region of interest was blood flow in the carotid, cerebral, and facial arteries during sleeping (SL), standing (ST), and mild head-down (HD) tilt conditions [10]. The initial variations in velocity were stabilized to steady state within few minutes from change of posture, however, the facial arteries in same time period were found to be unregulated. In another clinical study [11], five healthy male volunteers were subjected to various postures such as SL, HD, and head-up (HU) positions. It was concluded that the blood flow velocity in femoral and brachial arteries increased during postural change from SL to HD, while it decreased during change from SL to HU posture influenced by venous pressure. A part of the investigation also conducted on the changes in the structure and functioning of the carotid and femoral arteries during prolonged HD tilt in healthy volunteers [12]. The results revealed that volumetric flow increased in the carotid artery and decreased in the femoral artery, possibly depending on perfusion of vascular bed characteristics. A similar study [13] focused on orthostatic tolerance and evaluated the variation in various cardiovascular parameters due to changes in posture, such as HD tilt, supine, and HU tilt in male and female populations. It was noticed that these parameters had greater differences during HU than during HD shift. However, there was no significant effect of sex on any parameter. In another study [14], cerebral blood flow velocity and BP were evaluated in the supine and HU positions in non-stroke patients. Significant changes were observed in haemodynamic parameters during head change positions, which is useful in understanding and managing stroke patients. Further, such clinical studies have been performed in healthy individuals and extended to ischemic stroke patients; thus far, the results have demonstrated a decrease in arterial pressure and an increase in cerebral velocity during gradual changes in head position, such as supine, HU, and HD [15]. Initial changes in posture were assigned to supine and elevated head position, and the focus was on evaluating BP variability. The magnitude of this parameter in adverse stroke outcomes was greater in the elevated head position than in the supine position [16].

In addition to clinical studies, many recent studies have used an extensive mathematical approach to quantify the effect of altered gravity, especially related to space and microgravity applications. Experimental investigations are limited to providing a detailed understanding of the flow behavior during postural changes. Hence, to overcome these limitations, investigations have been performed to understand the mechanics of blood flow. Numerical model was developed by ref. [17] to understand the effect of intrathoracic pressure, intraventricular and intravascular hydrostatic pressure on cardiac functions. During supine, sitting (SI) and ST postures pressure variations in intrathoracic cavity and the effects of hydrostatic pressure were simulated for zero, one, and 1.8 G conditions respectively. In contrast to the zero G condition, a considerable increase in intrathoracic pressure was experienced with increasing gravity, resulting in 12% and 14% decreases in cardiac output for one and 1.8 G supine, respectively. A similar study was performed using a mathematical model to evaluate BP and flow velocity changes variation during the postural change from SI to ST by adopting two different types of control mechanisms: auto and autonomic-regulation [18]. In their study, one-dimensional (1D) mathematical model was established to study the effects of gravity on venous blood pooling during postural changes. The influence of postural changes on blood flow was further explored numerically by combining two different models of circulation (baroreflex model and lumped-parameter model) [19]. It was reported that, gravity directly affected the pulmonary circulation resulting in BP changes during postural change of HU tilt and reverse, while the change of posture to HU tilt was influenced by the venous system. Moreover, an attempt was made to provide a solution to the consistent problems faced by astronauts in overcoming pre- and post-flight adaptions. It was discussed that regular practice of sheershasana, a yogic posture, tunes the cardiovascular system to the pooling of blood in the head. It was demonstrated through clinically measured electrocardiogram, heart rate, and BP from different sets of volunteers with the help of a tilt table for different postures [20]. A review summarized the mechanisms of physiological body adaptation during long-duration space exploration [21]. In another review study [22], different effects of partial gravity on the human musculoskeletal, cardiovascular, and respiratory systems were summarized using data collected from previous space missions. These findings highlighted the drawbacks of immediate effects during exposure to partial gravity and its influence on the cardiovascular system. They also stressed the need to maintain the long-term health of astronauts through countermeasure exercises to overcome the drawbacks of partial gravity exposure. In another study [23], researchers summarized the knowledge gained in the adaptation of cardiovascular physiology and its functioning effects during short and long-duration microgravity exposure. It also highlighted various current countermeasures adopted to reduce the risk during space travel. A similar review provided an overview of the perception of humans to gravity and the adaption of different sexes to altered gravity [24]. This review study further emphasizes the need for extensive research to understand the dynamics of physiological adaption during different gravity levels. This review also proposes the need for artificial gravity and its importance as an alternative between short and very-long-arm centrifuges.

Although several analytical and clinical methods have been employed to demonstrate the effect of gravitation force on blood flow, there are quite a few numerical studies which have investigated and presented the importance of postural changes.

One such numerical study considering altered gravity conditions was on anatomically realistic Circle of Willis and Carotid bifurcation system under auto-regulation [25]. Significant changes in displacement of arterial wall and blood flow velocity were well established through numerical coupled field simulations during different posture such as ST, supine, and hand ST. Similarly, the influence of G force on cerebral circulation with stenosed internal carotid artery (ICA) was numerically investigated through simplified vasculature under different conditions [26]. The significant changes due to altered gravity conditions were observed in systemic and cerebral vascular blood flow through these numerical simulations.

In another previous study [27], the effect of posture change was adopted in a patient-specific pulmonary arterial model, and the researchers numerically investigated the variation in perfusion for different orientations of the lung. The results of different postures, such as upright, prone, and supine, demonstrated significant flow changes. However, there was a continual decrease in flow in the cranial and caudal regions for all postures. Furthermore, cardiovascular circulation system was numerically simulated [28], using 1D dissipative particle dynamics approach in healthy and stenosed subjects incorporating gravity, plasma and blood cells. They demonstrated the impact of atherosclerosis and influence of gravity component on circulatory system during different postures. A two-dimensional (2D) nonlinear blood flow model in a stenosed artery was evaluated [29] by adding gravity through finite difference approximations. Their initial work focused on understanding the flow dynamics demonstrated through the streamlines, axial velocity, and wall shear stress (WSS). Later, a few modifications were incorporated into the numerical equations to improve the accuracy of the results [30]. Further extension of this study revealed higher pressure and axial velocity, but lower WSS and recirculation regions in the presence of gravitational force [31]. Another study [32], investigated the flow patterns in the three-dimensional (3D) cervical artery under gravitational load. The inclusion of gravity led to a significant difference in the velocity distribution and shear stress distribution. The shear stress in the branches was higher and the flow velocity in the daughter vessel was lower than those in the parent vessel. Recently, a mathematical 1D-0D multiscale model was validated to understand the response of the cardiovascular system during long-term space travel in contrast to other conditions in gravity [33]. Cardiovascular parameters, including mean and pulse pressure, were reduced, thus augmenting the cardiac deconditioning scenario.

In previous clinical studies, understanding the influence of posture on blood flow was extensive in healthy volunteers and limited to high-risk stroke patients. At the same time, both experimentally and mathematically validated models have highlighted the importance of the cardiovascular system and its adaptation during altered gravity scenarios. Moreover, several studies have demonstrated the influence of different postures on the cardiovascular system through numerical simulation, but the majority of them investigated either 2D or 1D cardiovascular models, and patient-specific models involving flexible arteries are limited. Hence, it is necessary that there is a requirement to quantify haemodynamics and understand the influence of gravity during posture change in patient-specific cases. Such changes in flow behavior can be investigated using numerical tools and compared with FSI simulations. The present work is an extension of a previous fundamental study performed on idealistic carotid arteries with normal and stenosed conditions during changes in posture, such as SL, ST, and HD positions. Thus, the present study focuses on patient-specific cases of the carotid bifurcation system, and the variation in the haemodynamics behavior was investigated during changes in the postures. The patient-specific case consisted of internal carotid bifurcation diagnosed with a giant aneurysm on the left side, while the right side appeared to be normal. Numerical simulations were performed under physiological conditions using FSI, and the investigations of various postures such as SL, ST and HD positions in the right side internal carotid bifurcation (RICA) and left side internal carotid bifurcation (LICA) were compared.

## Methods

### Theory

In the present investigation, the blood flow in the carotid artery is assumed to be Newtonian, laminar, incompressible, and governed by the Navier–Stokes equations of incompressible flows [34]. The mass and momentum conservation governing equations for an incompressible fluid can be written in tensor notation as follows:

1 $$\nabla \nu =0$$

2 $$\rho \left(\frac{\partial \nu }{\partial t}+\nu \nabla \nu \right)=\nabla P+\nabla \tau +{g}_{i}$$

where $\rho$ is the density, $\tau$ is the stress tensor, $\upsilon$ is the velocity vector, $P$ is the pressure at time *t*, and the source term ${g}_{i}$ is added to Eq. (2) to take the gravitational effects into account.

The deviatoric stress tensor is related to the strain rate tensor, which is usually expressed as an algebraic equation in the following form [25]:

3 $$\tau \, \text{=}\upmu \left(\dot{\gamma }\right)\dot{\gamma }$$

4 $$\dot{\gamma \, }\text{=}\left(\frac{{\partial\upnu }_{i}}{\partial {x}_{j}}+\frac{{\partial\upnu }_{j}}{\partial {x}_{i}}\right)$$

where $\upmu \left(\dot{\gamma }\right)$ is the apparent viscosity, and $\dot{\gamma }$ is the shear rate. As discussed in refs. [35, 36], the modified momentum equation suitable for two-way FSI is adopted in addition to the continuity equation, as shown in Eq. (5).

5 $$\frac{\partial }{\partial t}\underset{\Omega }{\overset{}{\int }}\rho \partial\Omega +\underset{\mathrm{S}}{\overset{}{\int }}\rho \upsilon -{\upsilon }_{b}.n\partial S= {\int }_{S}{\tau }_{ij}{i}_{j}-P{i}_{i}.n.\partial S+{\int }_{\Omega }{b}_{i}\partial\Omega$$

where $\rho$ is the density, $\tau$ is the stress tensor, $\upsilon$ is the velocity vector, ${\upsilon }_{b}$ is the grid velocity, $P$ is the pressure, ${b}_{i}$ is the body force comprising the gravitational force ${g}_{i}$ at time *t*. With the reference pressure at the heart level, the BP gradient due to the height difference is added to the applied boundary condition. It is assumed that the heart should deliver the same amount of blood under altered gravity [9]. Further, physiological importance of BP and the effect of its components on blood vessels has been reported in ref. [34]. In the present study [34], the arterial wall is assumed to be linear elastic, incompressible, isotropic and homogenous in nature. ANSYS structural solver captures the transient behavior of elastic artery as described in Eq. (6). The stiffness matrix is updated in each time step using Newmark method, while direct solver for every time step [37].

6 $$\left[M\right]\left\{\ddot{U}\right\}+ \left[C\right]\left\{\dot{U}\right\}+ \left[K\right]\left\{U\right\}= \left\{{F}^{a}\right\}$$

where $M$ is the structural mass matrix, $C$ is the structural damping matrix, $K$ is the structural stiffness matrix, ${F}^{a}$ is the applied load vector, $\ddot{U, }\dot{U}, U$ represent the acceleration, velocity, and displacement vector, respectively [2, 17].

**Fig. 1:**
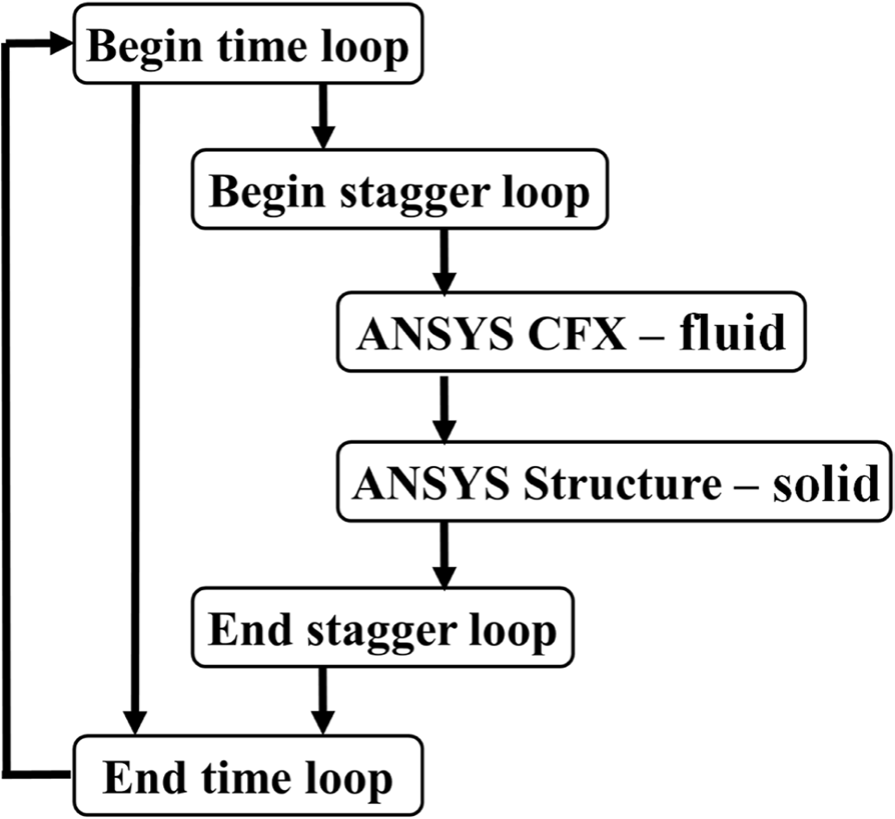
FSI algorithm

FSI transient simulation is carried out by considering FSI solver in ANSYS 17.0 adopting two-way sequential coupling strategy. As mentioned in Fig. 1, the FSI solver in ANSYS solves the solid and fluid domains individually using STRUCTURE and CFX, respectively. Initially, the fluid domain will be solved and the resulting pressure will be applied to the solid domain through the interface. The pressure load at the interface will be input load for solid domain solution and further details can be found in refs. [34, 37].

### Modelling and analysis

**Fig. 2:**
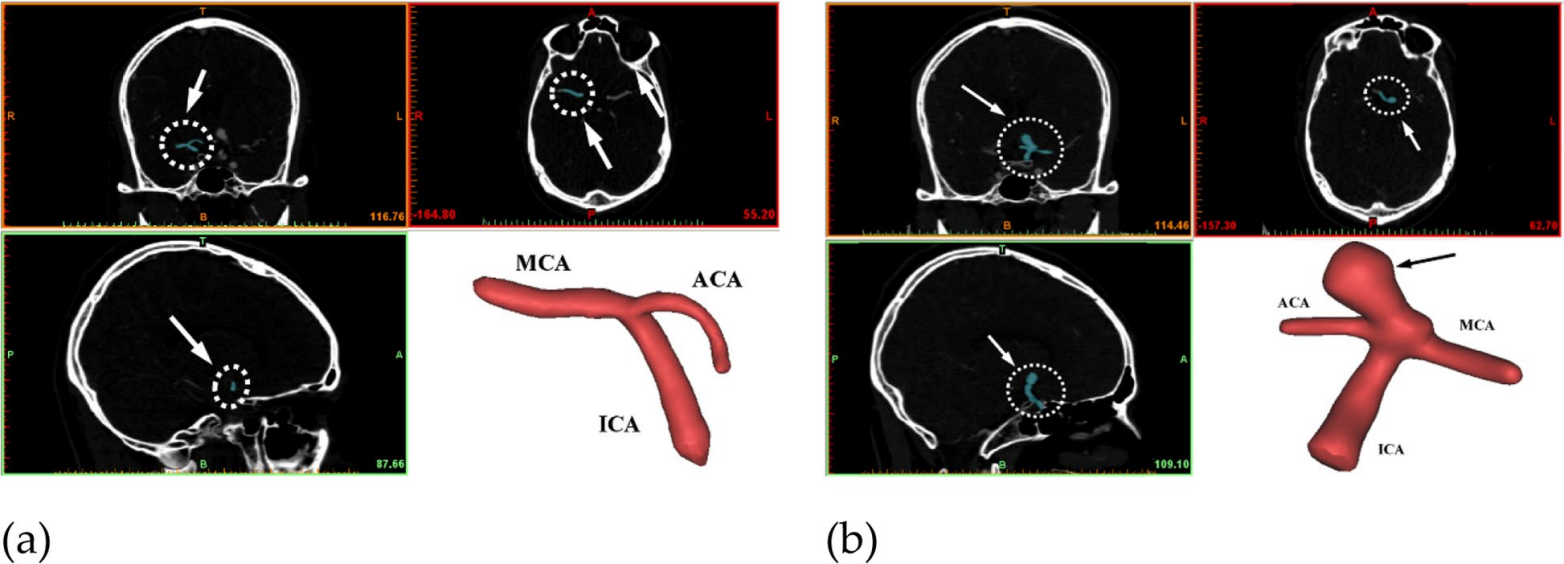
Different views of CT scan of ICA bifurcation. **a**: Normal; **b**: Giant aneurysm

**Fig. 3:**
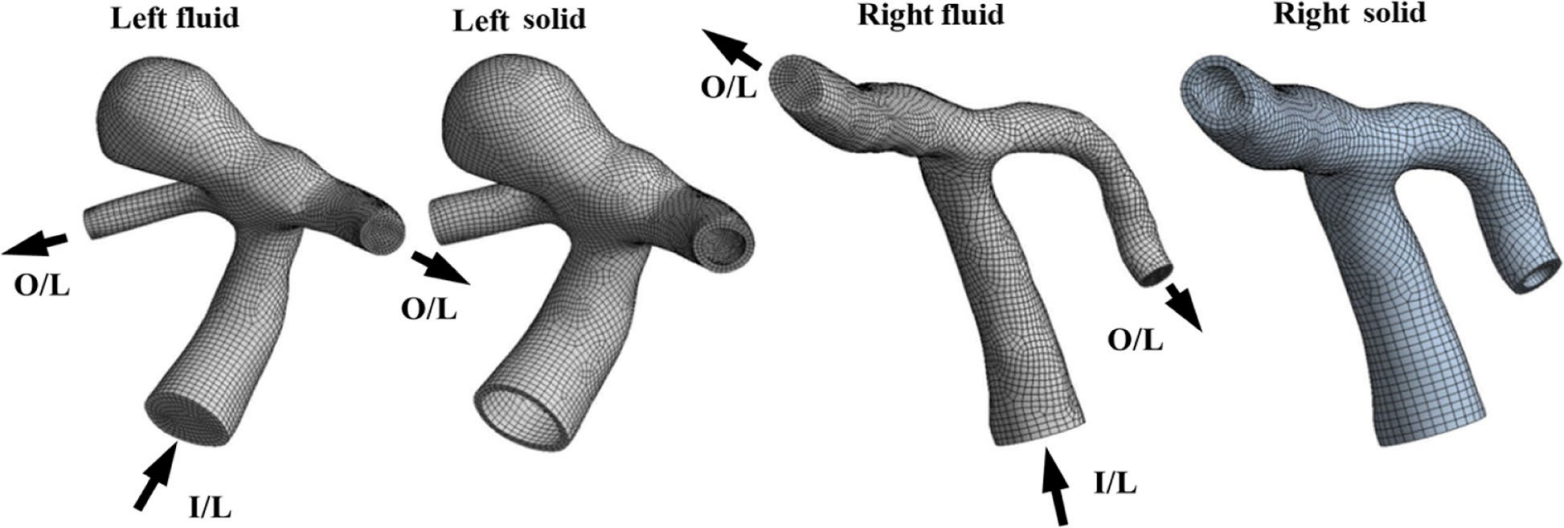
Carotid bifurcation FSI models (left and right side). The left side has giant aneurysm at the ICA bifurcation and the right side is normal

The present study is a patient-specific case study of a 58-year-old female patient who had reported with a hemorrhagic stroke (sudden subarachnoid hemorrhage due to aneurysmal rupture) with a broad necked aneurysm arising from the bifurcation of the left ICA, while the right ICA bifurcation appeared to be normal. The location of the broad neck aneurysm and normal ICA bifurcation is highlighted in three different views, as shown in Fig. 2. The grid sizes of the 3D FSI models for the LICA as shown in Fig. 3 consist of 35230 and 21270 hexahedral elements for the fluid and solid models, respectively, while the RICA consists of 36480 and 22450 hexahedral elements for the fluid and solid models, respectively.

**Fig. 4:**
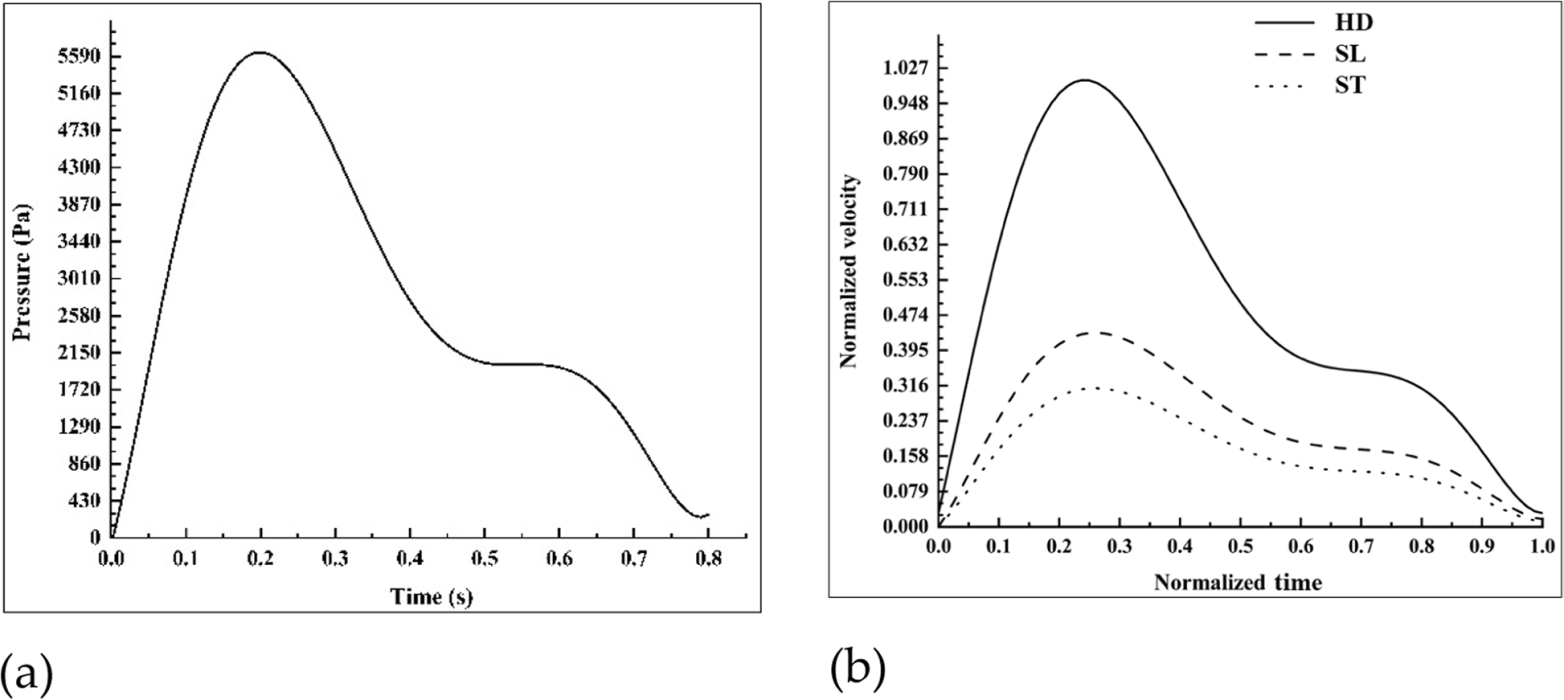
Various applied waveforms in the fluid domain. **a**: Outlet pulse pressure waveform; **b**: Inlet velocity waveform for different postures

The pressure waveform in a typical carotid artery bifurcation is in the range 110–120/70–80 mmHg, as per clinical observation [38]. However, assuming the peripheral resistance from the arteries in downstream side, a pulsatile pressure waveform was applied at the outlet, as shown in Fig. 4(a) [39, 40]. The velocity spectrum obtained from patient specific ultrasound carotid Doppler was considered as pulsatile velocity waveform at the inlet, as shown in Fig. 4(b) [38, 39]. In the present study, focus is on understanding the haemodynamics for three conditions, that is, SL, ST and HD positions. Hence, the applied numerical boundary conditions at inlet and outlet should follow physiology of postural changes. Therefore, effect of gravity on blood flow during SL is nil, however to counter the gravity, during ST and HD position, 1G and -1G force is applied downwards and towards the head respectively [20, 25]. Hence, considering these assumptions, the pulsatile velocity waveform (normalized) as shown in the Fig. 4(b) is applied at inlet for various postures [25, 34].

The considerable increase in the flow is at peak systole, particularly during the HD position [34]. Thus, in the present investigation of transient FSI analysis, laminar flow is adopted in the SL and ST posture, while the k–ω turbulence model (medium intensity factor) is prescribed for the HD posture (Reynolds number is greater than 3000). The flow properties of viscosity and density are assumed to be 0.004 Pa and 1060 kg/m^3^, respectively. The elastic properties of arterial wall assumed to have Youngs modulus of 5 × 10^5^ Pa, density of 1200 kg/m^3^ and Poisson’s ratio of 0.48 [39, 41]. The entire simulation was performed for five pulse cycles, and each pulse cycle (0.8 s) was discretized into 250-time steps and the results captured during the last cycle was presented. Although the physiological influence of postural change during transition time is quite complex, however the stabilized flow behavior due to autoregulation is assumed in the present investigation.

## Results and discussion

**Fig. 5:**
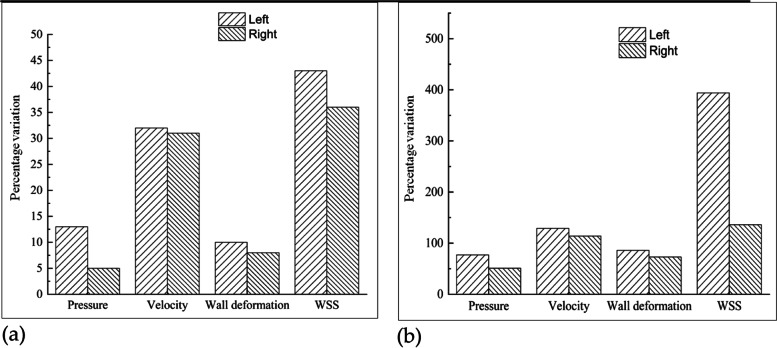
Percentage variation during change of posture. **a**: From SL to ST; **b**: From SL to HD

The haemodynamic parameters were evaluated during HD, ST and SL postures on both normal and aneurysm side of patient specific carotid system. These parameters include velocity, WSS, pressure, deformation and von-Mises stress in arterial wall. The changes in the flow behavior observed at peak systole are shown in Table 1 during the change of different postures. The flow variables in the left ICA bifurcation with the aneurysm were higher than those of the normal ICA bifurcation on the right side. The aneurysm side has a large pressure variation in contrast to the normal side during postural change from SL to HD position when compared to the change from SL to ST. The percentage variation of these flow variables is shown in the Table 1 and similar characteristics are depicted in Fig. 5. Compared to the pressure, the velocity variation in addition to the WSS and wall deformation on the normal side showed a higher percentage change than the aneurysm side. The differences in flow behavior are described individually in subsequent sections.

**Table 1:** Assessment of haemodynamics parameters observed in aneurysm and normal side ICA bifurcation during different postures

Flow variables	SL to ST		SL to HD	
	**Left**	**Right**	**Left**	**Right**
Velocity change (m/s)	0.455	0.265	1.649	1.400
WSS change (Pa)	10.217	2.809	38.115	26.029
Pressure change (Pa)	890.58	330.08	5525.17	3112.35
Wall deformation change (mm)	0.113	0.020	0.841	0.227
von Mises stress (Pa)	13079	91396	1509	14427

### Velocity

**Fig. 6:**
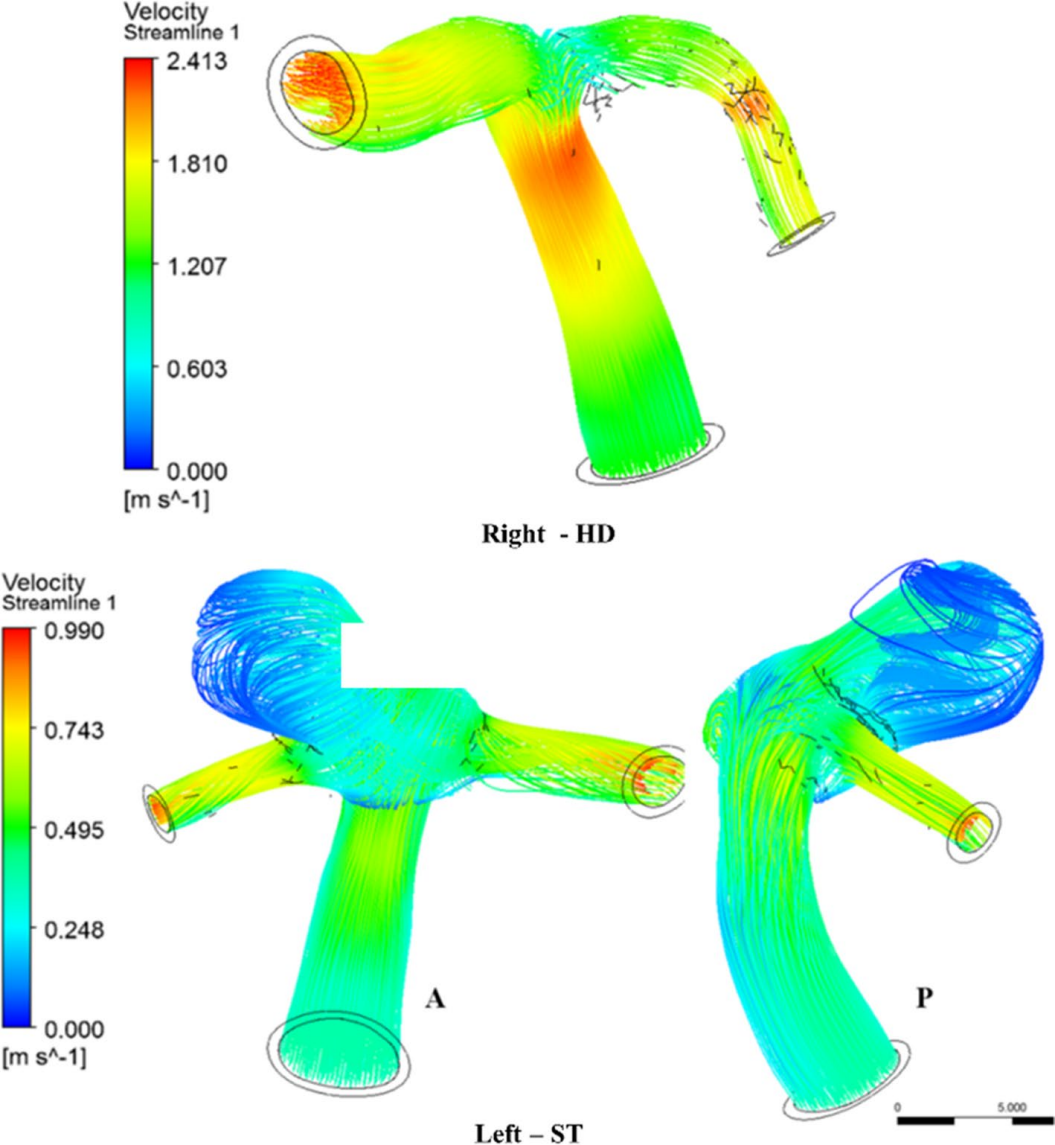
Comparison of velocity streamlines during peak systole

The streamlines of velocity normal and aneurysm ICA bifurcation are described in Fig. 6 during peak systole for HD and ST postures, respectively. In the normal ICA bifurcation, the upstream of the bifurcation has an intense flow velocity, which impinges on the arterial wall; this is then reduced at the bifurcation tip owing to the flow distribution and is further stabilized in the downstream end of the middle cerebral artery (MCA) and anterior cerebral artery (ACA). On the aneurysm side, the flow velocity enters the dome at a higher velocity, as seen in the anterior view (A), swirls in the sac, and exits, as seen in the posterior view (P). The velocity will be stagnant at the core of the sac, as the complete flow exit chances are less due to the pulsatile nature of the flow [42, 43].

**Fig. 7:**
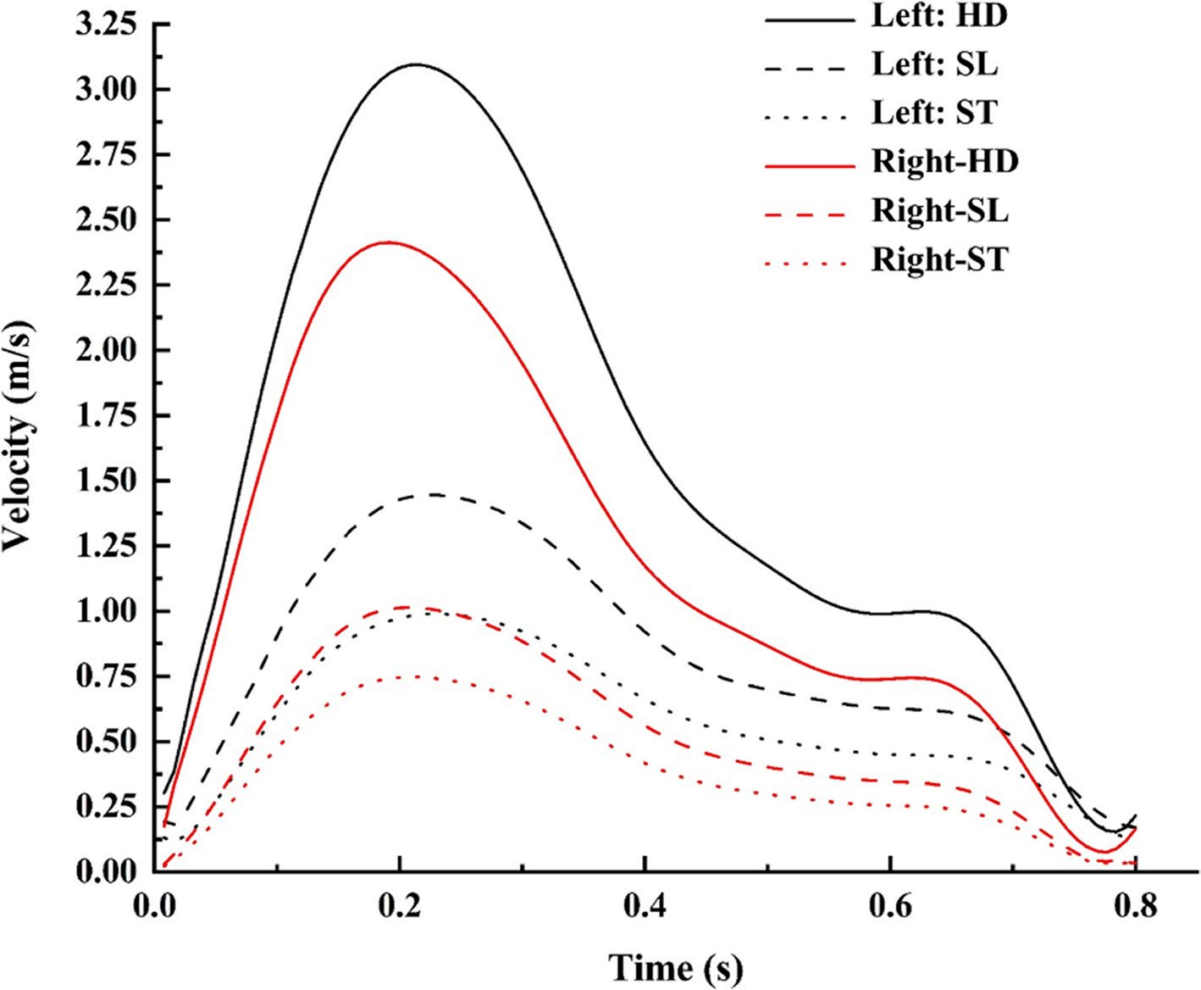
Maximum velocity comparison for various postures

The flow in the downstream end of the aneurysm side is highly intense and disturbed, with the majority of flow entering the MCA as compared with the ACA, in contrast to the normal ICA bifurcation [6]. The high flow velocity entering the sac of the aneurysm elongates the dome towards the apex. Thus, a higher velocity is observed in the aneurysm than in the normal side for various postures. The velocity behavior during the various postures is shown in Fig. 7.

The flow velocity decreased by 30%, with a minor difference between normal and aneurysm models during the change of posture from SL to ST [Fig. 5(a)], while the change in posture from SL to HD has a significantly larger increase, as observed in Fig. 5(b). The flow swirling increases in the HD position, causing an increase in sac pressure and enlargement of the sac with a larger region of flow recirculation [44]. Pathologically, these large recirculation zones are dangerous as they increase the blood residence time and may induce blood clot or thrombus formation inside the aneurysm [45]. The newly formed fragile blood clots may exit the aneurysm with a potential risk of embolic stroke further downstream.

### WSS

**Fig. 8:**
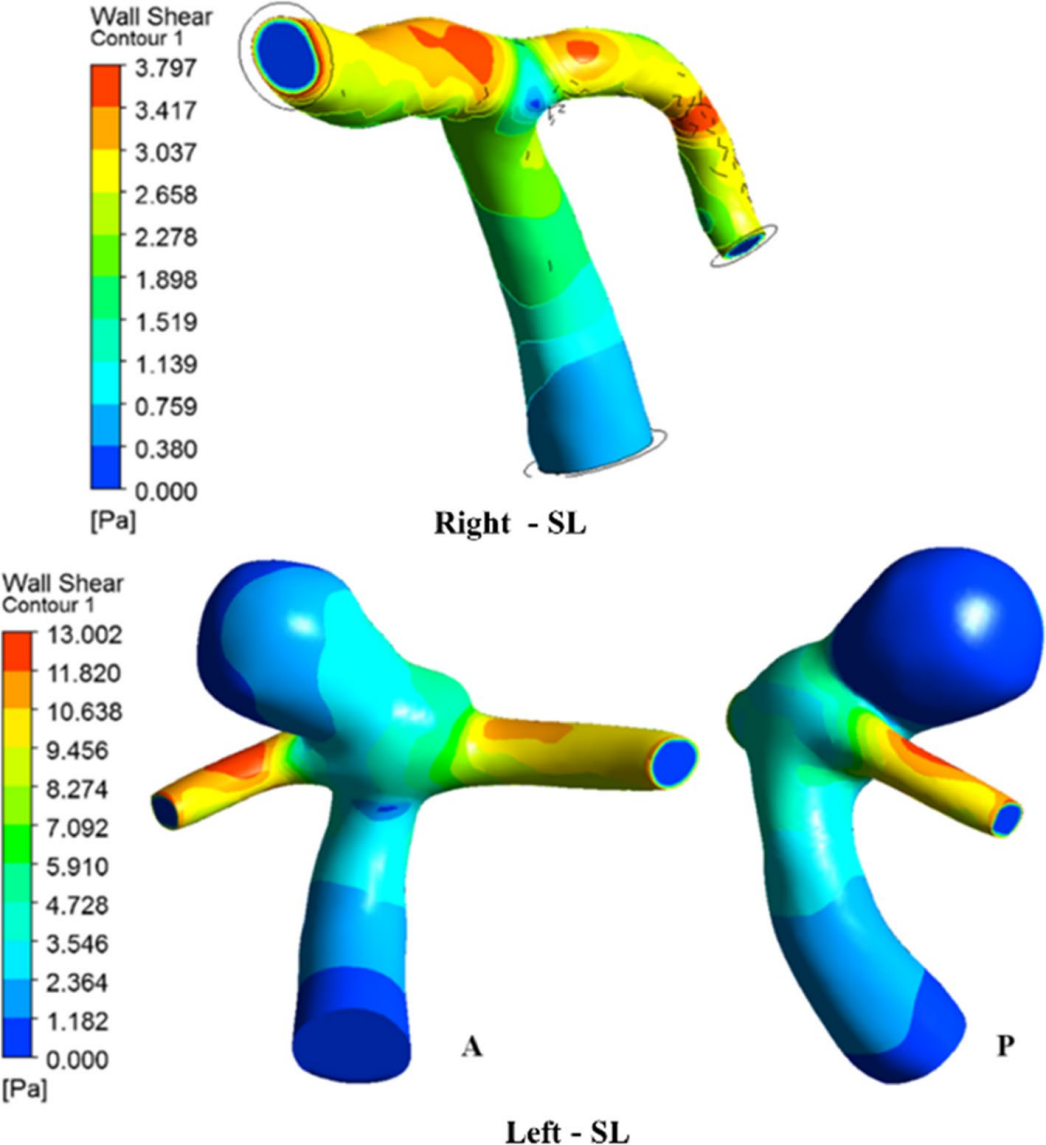
Comparison of WSS contours during peak systole

The most interesting haemodynamic parameter in relation to aneurysm progression is the WSS, which varies due to the pulsatile nature of the flow. The WSS value reaches to be maximum at peak systole when the inflow into blood vessel is maximum. The WSS distribution was found to be higher in the normal ICA bifurcation region, especially along the outer wall of the MCA and ACA, as shown in Fig. 8, during peak systole. The WSS was reduced at the bifurcation tip because the velocity is lower owing to the flow jet from the ICA impinging at the bifurcation tip region. The WSS distribution in ICA aneurysm is the highest in the neck region and lowest in the dome region. The entry region of the sac has a maximum distribution of WSS, where the flow is the highest, especially at the neck region of the sac, while later, as the flow reduces towards the exit side of the sac, the WSS also drops gradually.

**Fig. 9:**
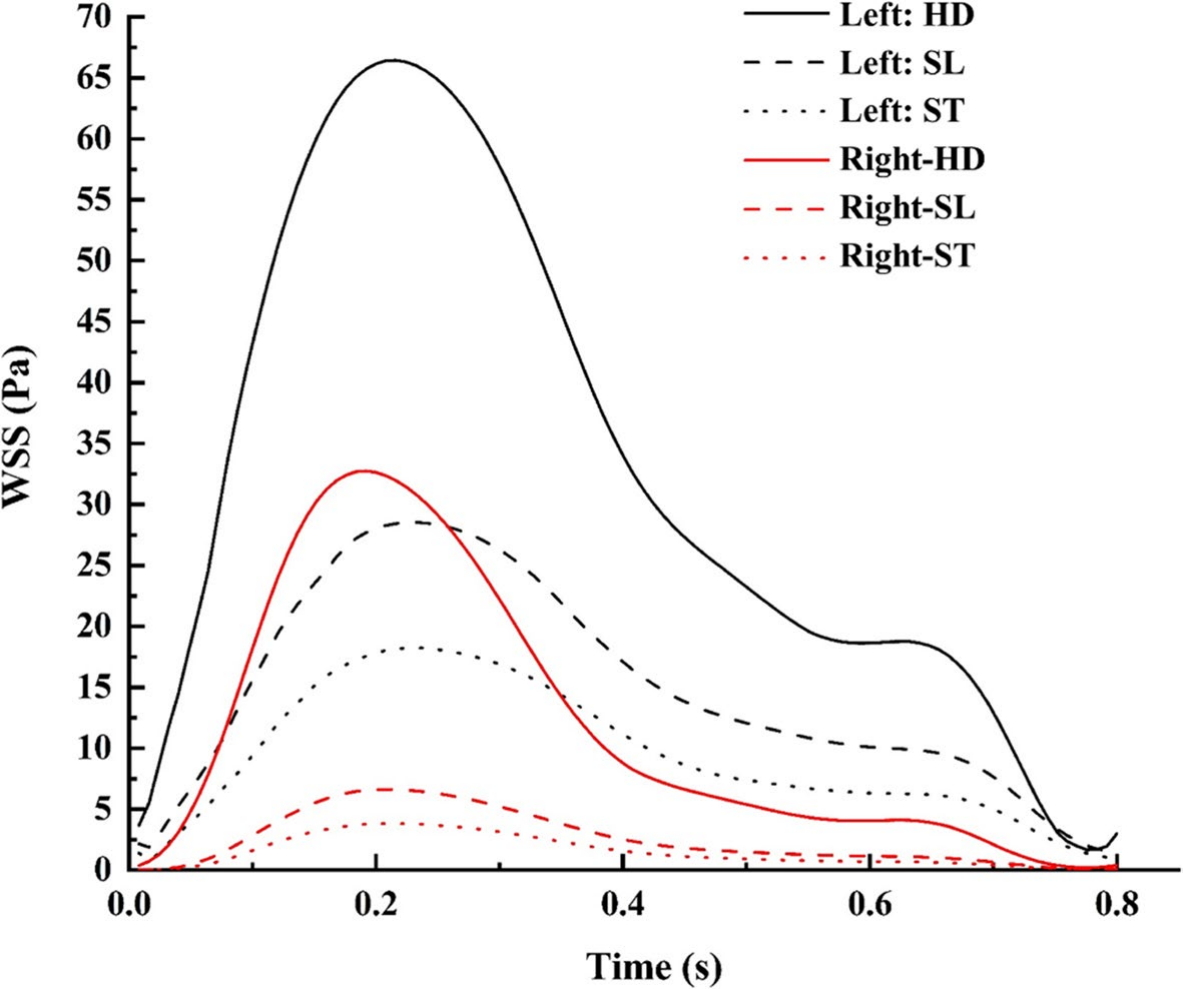
WSS comparison for various postures

The range of WSS in the dome is relatively low and within the lower limit of the physiological WSS range [6]. This lower range of the WSS located near the apex may also play an important role in aneurysm rupture through degradation of the aneurysmal wall because of the abnormal metabolic activity in the arterial wall and aggregation of inflammatory cells in the region due to relative flow stasis [46]. This separate mechanism of endothelial injury in areas of low WSS also has the potential to cause further atherosclerotic disease progression [47]. Another interesting finding is that high WSS near the neck of the sac results in lamina loss, media thinning, and bulge formation, thus initiating aneurysm formation. The initial enlargement of the aneurysmal bulge results in flow recirculation within the sac; thus, the flow behavior is dominated by low WSS. A low WSS will further lead to wall inflammation and accentuate thrombus formation [48–50]. Moreover, the maximum WSS was observed in the aneurysm case compared to the normal ICA bifurcation, as shown in Fig. 9. The flow jet at the tip of the normal ICA bifurcation is more intense in the HD position than in the ST or SL positions. In the aneurysm case, sac filling was more intense in the HD position than in the ST or SL positions. The WSS was distributed to nearly half of the dome in the SL and ST positions, but in the HD position, it covers almost the entire dome region. There is a large difference in flow parameters compared to the normal bifurcation owing to the presence of an aneurysmal sac. The postural change from SL to ST reduces the WSS by 35% and 45% in normal ICA and ICA with aneurysms, respectively [Fig. 5(a)]. In the HD position, the WSS increases by ten times in the aneurysm ICA and three times in the normal ICA bifurcation, as shown in Fig. 5(b). Thus, the HD position (or any other cause of increased flow or pressure) should be avoided, because the sudden increase in WSS and higher sac pressure may accentuate the risk of aneurysm rupture and accelerate atherosclerotic disease progression.

### Pressure

**Fig. 10:**
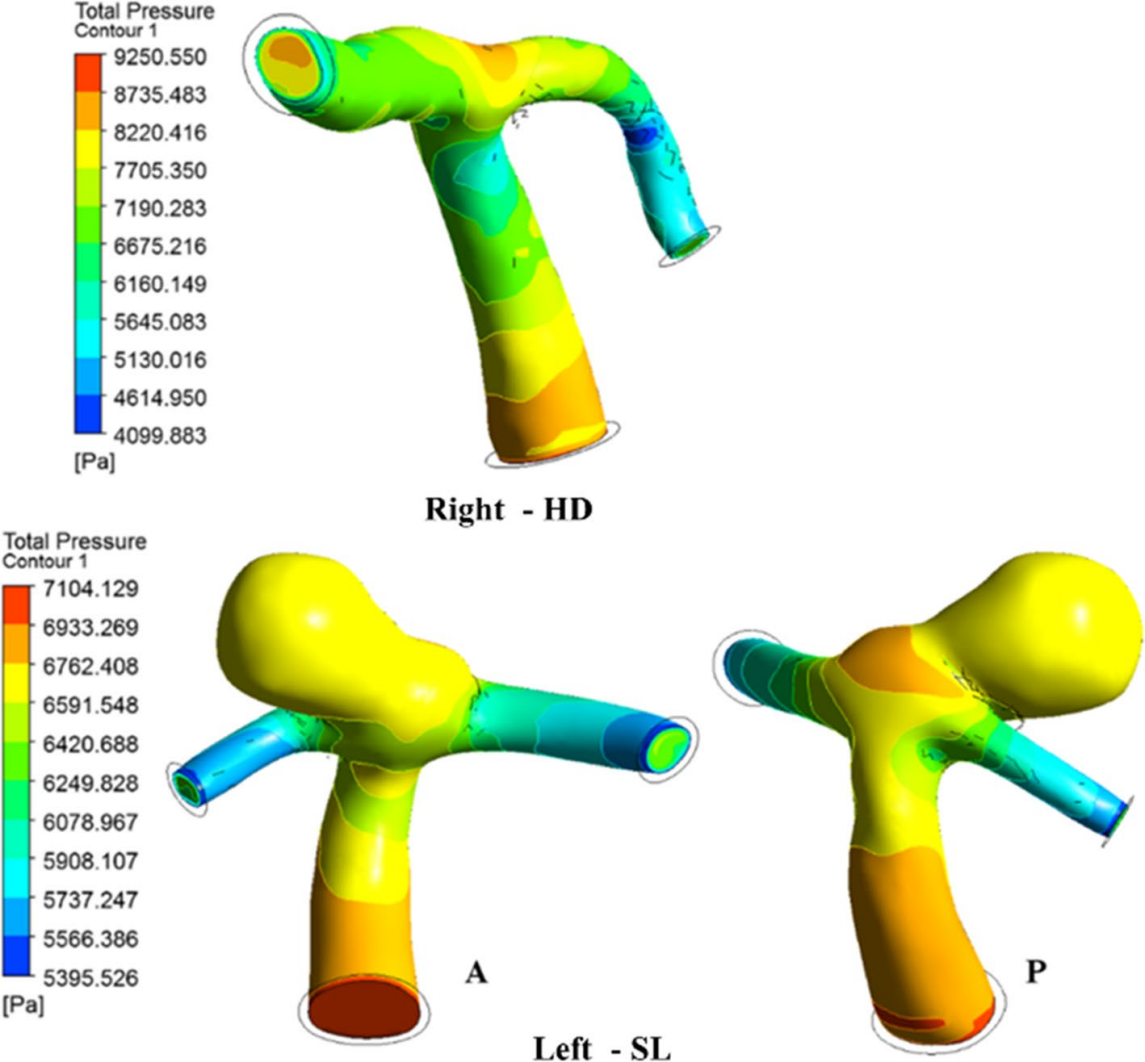
Comparison of pressure contours during peak systole

**Fig. 11:**
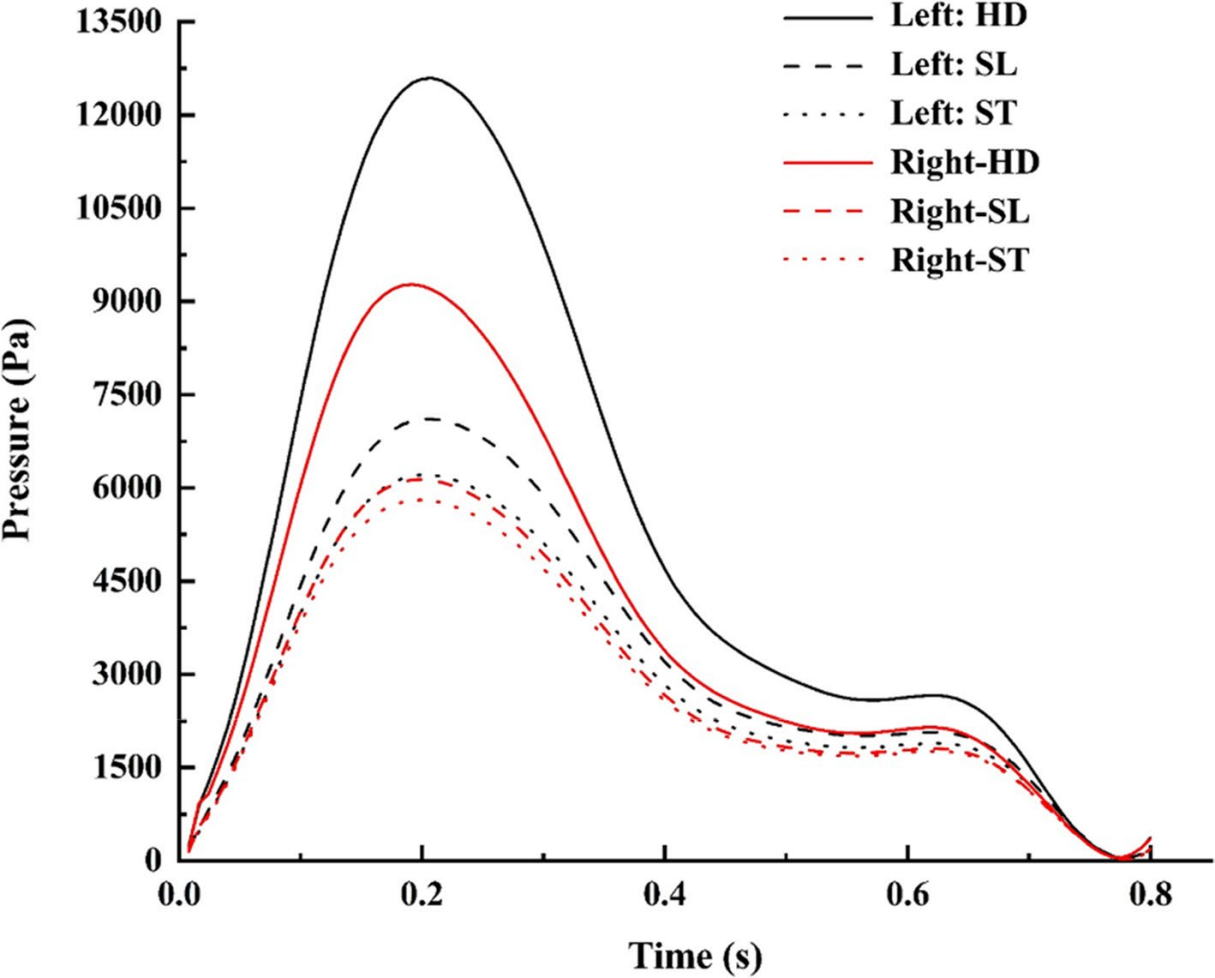
Pressure comparison during various postures

The pressure distribution in the aneurysm ICA bifurcation was higher than that in the normal ICA bifurcation. On the normal side, the pressure at the bifurcation tip increases as the flow velocity dips and distributes into the MCA and ACA. In the case of aneurysm ICA bifurcation, the entry side of the dome is subjected to a higher pressure when compared to the rest of the dome, as shown in Fig. 10. The large pressure within the sac influences the inflation of the sac towards the apex [1, 6, 45]. The flow entry of the aneurysm can be seen in the P view, with a larger patch of higher pressure than the rest of the dome, as observed in the A view. Figure 11 compares the normal and aneurysm ICA bifurcation pressure variations for different postures.

During the HD position, the aneurysm side experiences a higher pressure than the normal side; even for other postures, such as ST and SL, the aneurysm side has a higher pressure than the normal side. During the change of posture from SL to ST during peak systole, the pressure drops by 5% and 12% in normal and aneurysm ICA bifurcation, as observed in Fig. 5(a) and Table 1. During the postural change from SL to HD, the pressure increases by 80% and 50% in the aneurysm and normal ICA bifurcation, respectively, as shown in Fig. 5(b).

### Arterial wall deformation

**Fig. 12:**
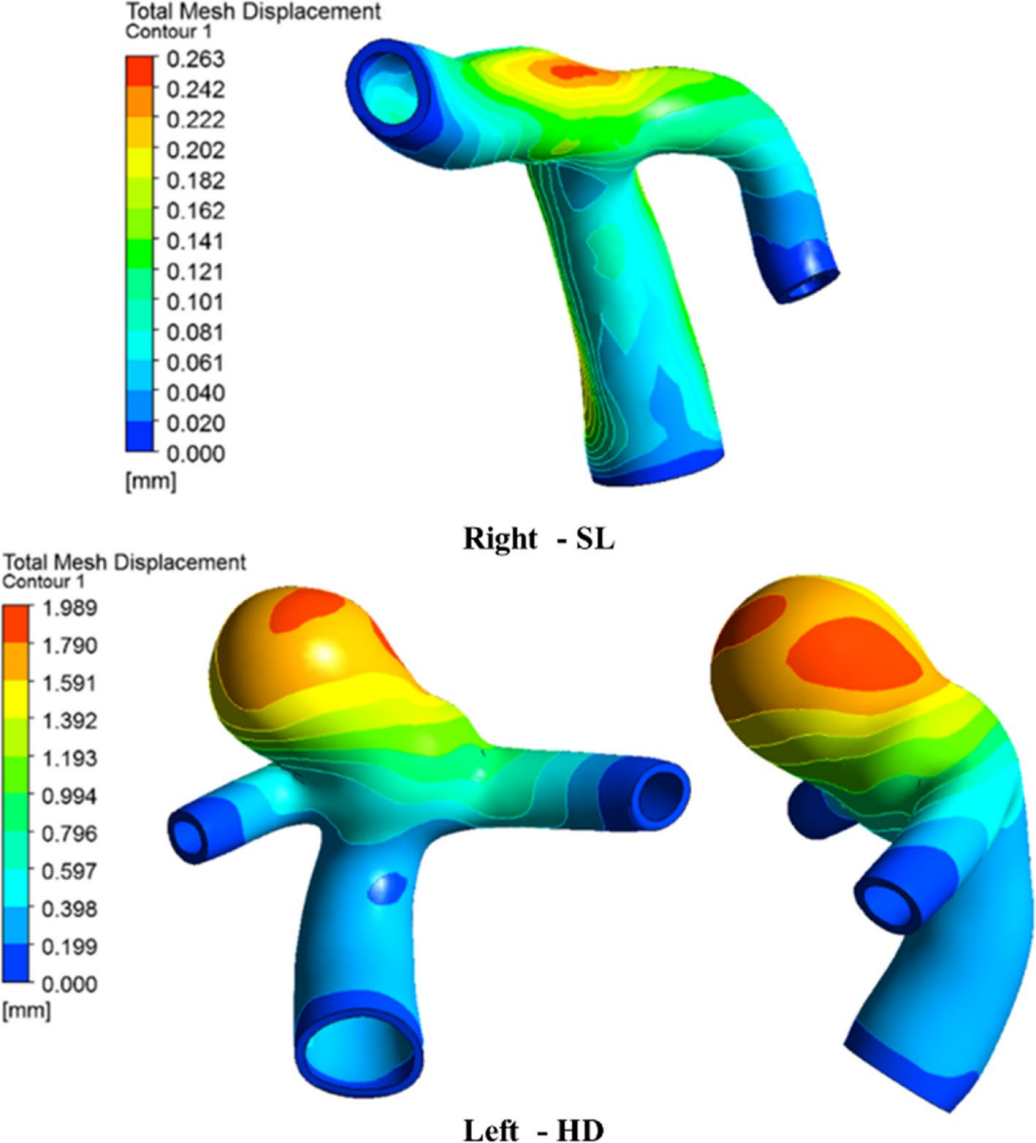
Comparison of wall deformation contours during peak systole

Figure 12 shows the wall deformation during peak systole in the normal and aneurysm ICA bifurcations. On the normal side, the maximum wall displacement is found to be at the apex of ICA bifurcation, which has a higher-pressure distribution [6]. The flow jet directly hits the apex because of the broad neck aneurysm, and most of the blood flow at the center of the sac is almost stagnant.

**Fig. 13:**
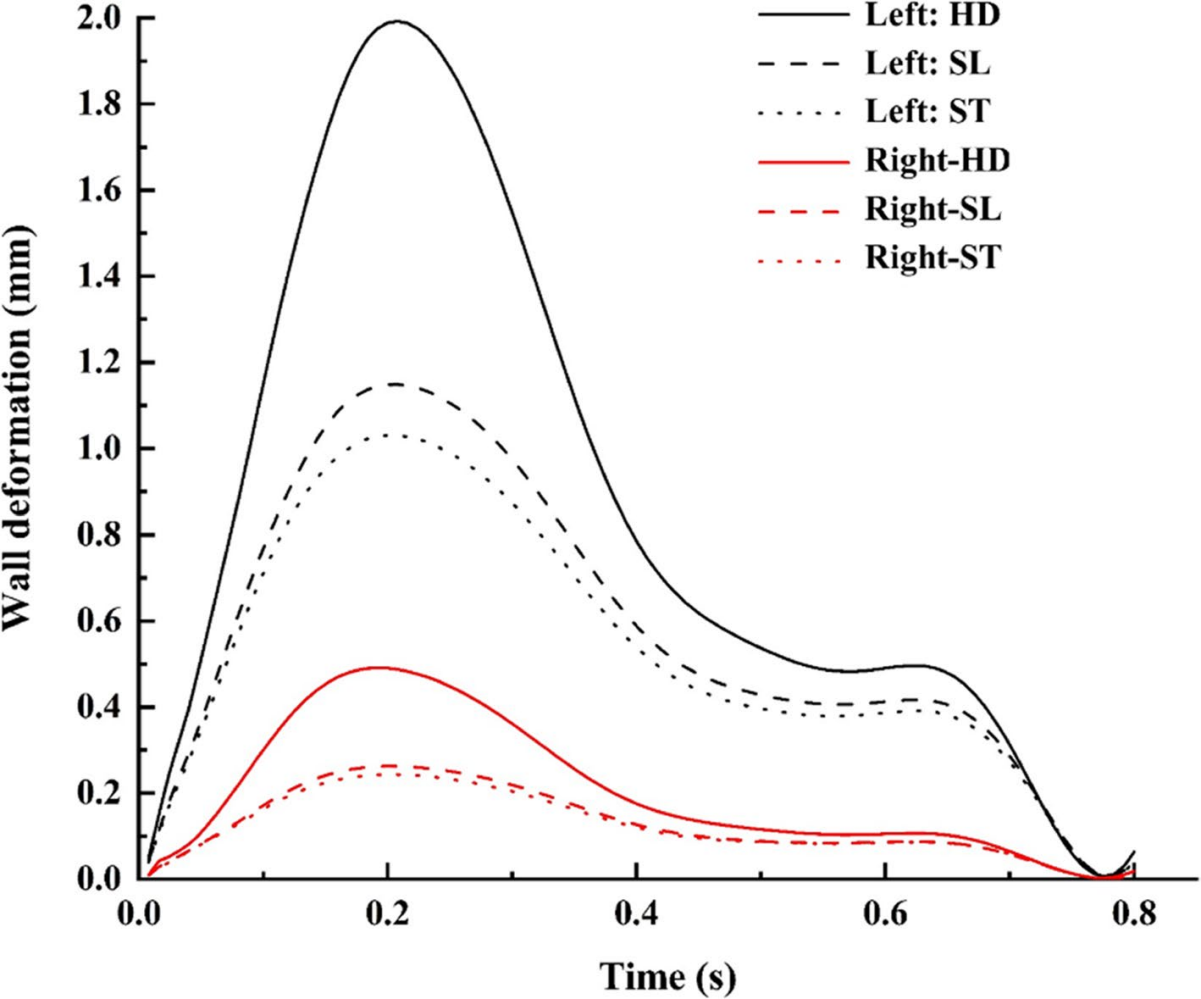
Wall deformation comparison for various postures

Hence, the entire sac is subjected to higher pressure; the apex region, including the entry side of the dome, experiences maximum deformation, and gradually decreases towards the neck region [51, 52]. The aneurysm side had a maximum deformation in contrast to the normal side ICA bifurcation. The wall deformation was compared for various postures, as shown in Fig. 13. The deformation decreases by 10% in the aneurysm while, its 7% in normal side [Fig. 5(a)] during postural change from SL to ST. However, there was a significant increase during the postural change from SL to HD in contrast to ST, with a major difference of 25% between normal and aneurysm sides, as depicted in Fig. 5(b).

### Arterial structural stress

**Fig. 14:**
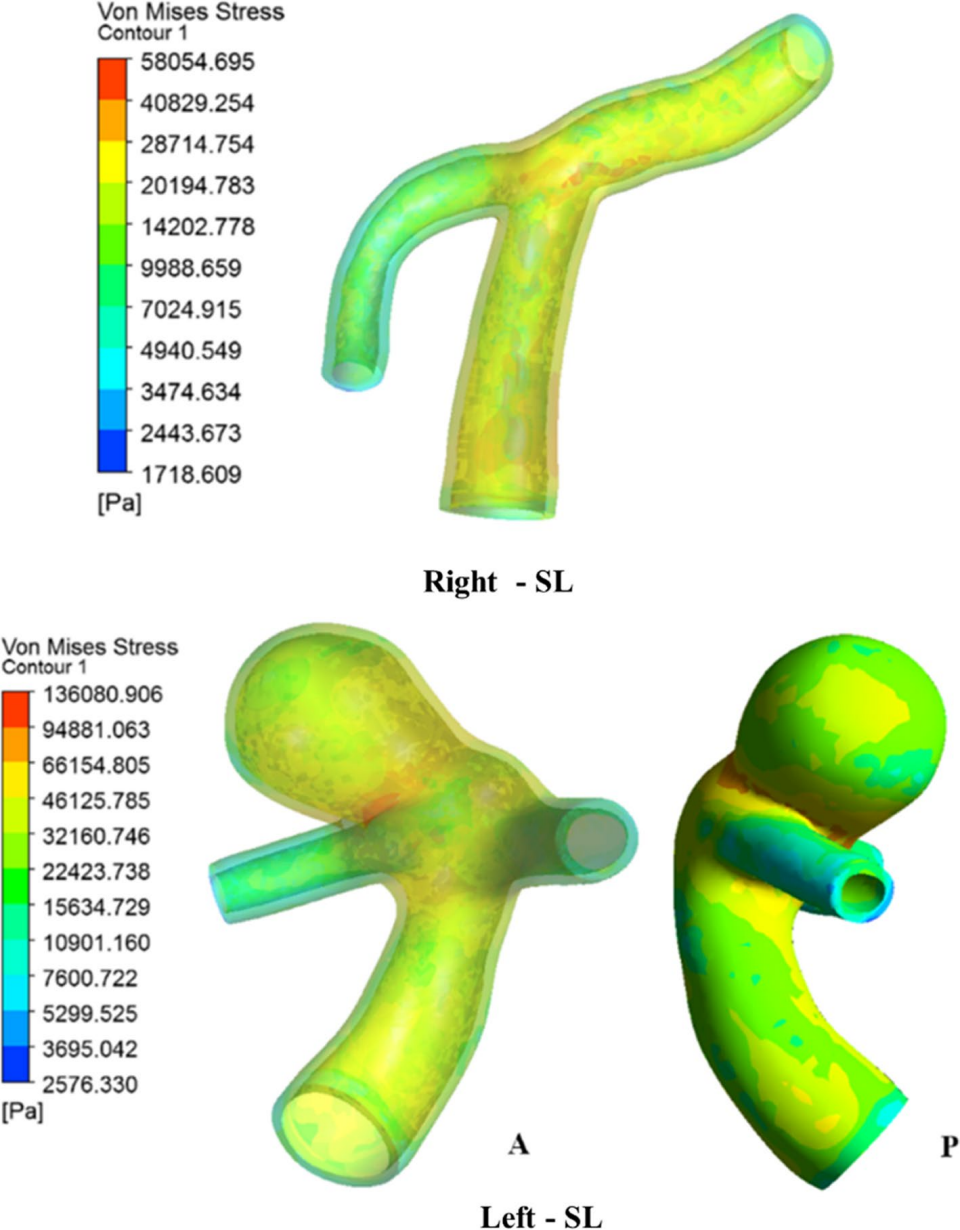
Comparison of von Mises stress contours during peak systole

**Fig. 15:**
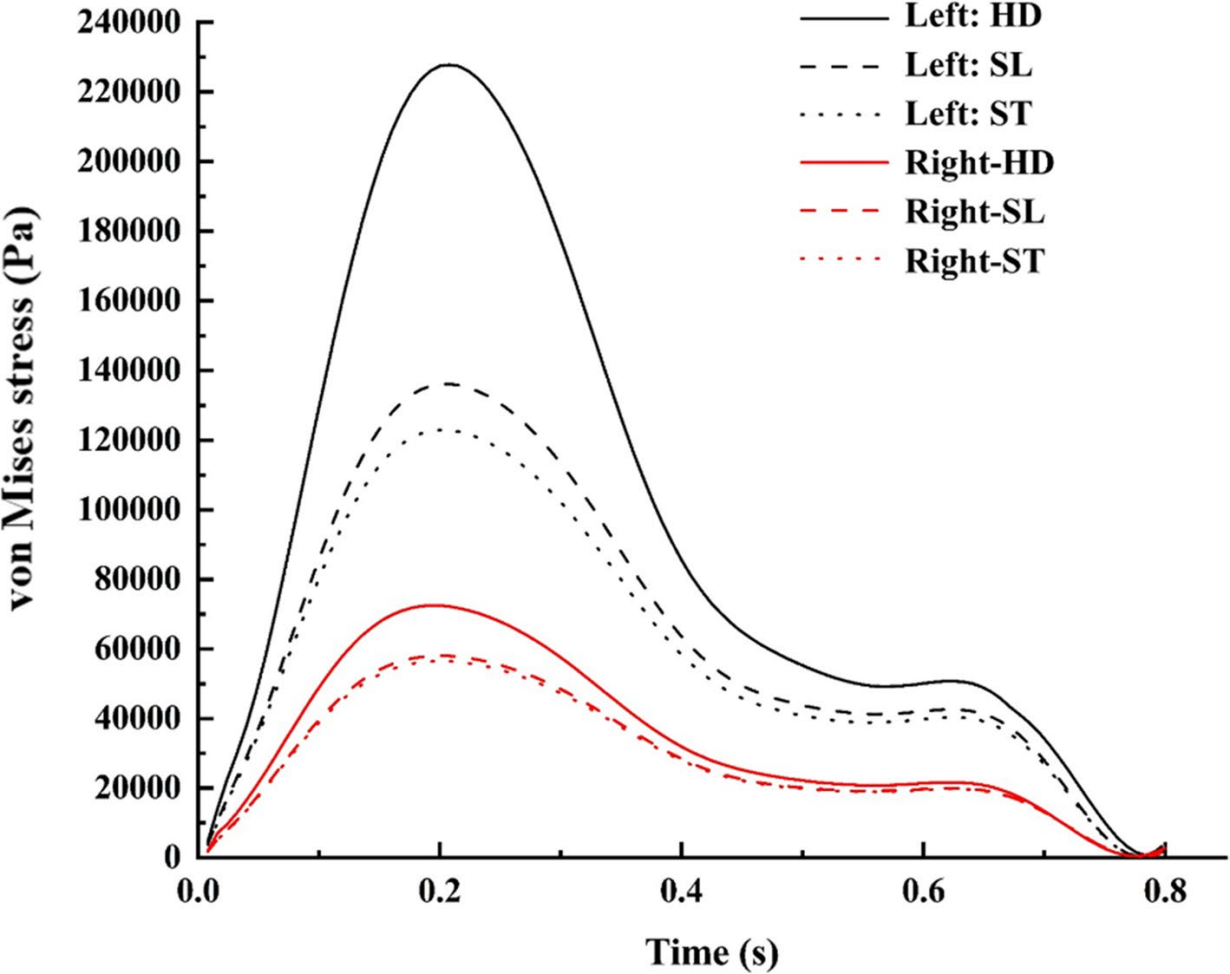
von Mises stress comparison for various postures

The von Mises stress is shown at peak systole in Fig. 14 in the right and left ICA bifurcation during the SL position. In normal ICA bifurcation, the maximum stress is found to be at the bifurcation tip, which has a relatively higher pressure [48]. In addition, moderately less stress was found to be distributed in the ICA and MCA, unlike the ACA. In the case of a broad neck aneurysm, a higher-pressure load allows for the deformation of the entire sac, resulting in intense stretching of the dome about the neck [3, 4]. Hence, this stretching of the dome neck causes maximum stress, especially at the entry side of the dome, as observed in the posterior and anterior views (Fig. 14) [49, 53, 54]. The stress concentration factor in normal and aneurysm ICA bifurcation was 2.92 and 4.80 respectively. The variation of von Mises stress in both the normal and aneurysm sides is shown in Fig. 15. The right-side aneurysm ICA bifurcation has a higher stress variation when compared with the left-side normal ICA bifurcation.

During the change in posture from SL to ST, the maximum stress dropped by 70% on the aneurysm side and 10% on the normal side, as shown in Fig. 5(a). Moreover, the maximum stress increases by 2.5% and 25% in the normal and aneurysm ICA bifurcations, respectively [Fig. 5(b)].

## Conclusions

The present investigation highlights the significant changes in the flow behavior in subject-specific cases of internal carotid bifurcation in different postures, with the ST posture as a reference. The investigation of flow behavior in this study provides a good comparison of haemodynamic changes under different categories of normal and diseased states. The obtained results indicate severe changes in HD position compared to the minor variations in ST position with reference to SL position. The WSS was found to have the most significant variation during postural changes. RICA demonstrates the common flow distribution observed clinically with fairly low flow separation and escalated pressures at the tip. The velocity and WSS decreased by 32% and 35% (LICA) to 45% (RICA), respectively, while the pressure and arterial wall deformation decreased by 13% (LICA) to 5% (RICA) and nearly 10% (in both LICA and RICA), respectively, during the ST posture. However, during the HD position, the velocity increased abruptly by more than four times (LICA and RICA) and the WSS by four times (LICA) to ten times (RICA). A high WSS value at the neck of the aneurysm and correspondingly low levels at the apex will induce thrombus formation, while the complex swirling flow and increased pressure in the dome increase the risk of rupture. In addition, the pressure increased by more than 80% (LICA) and 50% (RICA), while the wall deformation increased by more than 80% (LICA and RICA). The von Mises stress decreases by 70% (LICA) and 10% (RICA) during ST, while it increased by 25% (LICA) and 2.5% (RICA) during the HD posture. The maximum von Mises stress observed at the neck of the aneurysm also influences aneurysm rupture. RICA demonstrates the typical flow pattern observed clinically with mild flow separation and increased pressure at the point of bifurcation. A marginal increase in WSS closer to the bifurcation point was also observed (one of the reasons for aneurysm formation). The complex spiral flow and higher pressures prevailing within the dome accelerate the potential for aneurysm rupture.

## Data Availability

All data generated or analyzed during this study are included in this published article.

## Abbreviations

LICA: Left side internal carotid bifurcation
RICA: Right side internal carotid bifurcation
WSS: Wall shear stress
HU: Head-up
HD: Head-down
SL: Sleeping
ST: Standing
SI: Sitting
ICA: Internal carotid artery
BP: Blood pressure
FSI: Fluid-structure interaction
MCA: Middle cerebral artery
ACA: Anterior cerebral artery
1D: One-dimensional
2D: Two-dimensional
3D: Three-dimensional
P: Posterior
A: Anterior
